# A database of multi-channel intramuscular electromyogram signals during isometric hand muscles contractions

**DOI:** 10.1038/s41597-019-0335-8

**Published:** 2020-01-08

**Authors:** Nebojsa Malesevic, Anders Björkman, Gert S. Andersson, Ana Matran-Fernandez, Luca Citi, Christian Cipriani, Christian Antfolk

**Affiliations:** 1 Department of Biomedical Engineering, Faculty of Engineering, Lund University, Lund, Sweden; 2 Department of Translational Medicine - Hand Surgery, Skåne University Hospital, Lund University, Lund, Sweden; 3 Wallenberg Centre for Molecular Medicine, Lund University, Lund, Sweden; 4 Department of Clinical Neurophysiology, Skåne University Hospital, Lund, Sweden; 5 Department of Clinical Sciences in Lund – Neurophysiology, Lund University, Lund, Sweden; 6 School of Computer Science and Electronic Engineering, University of Essex, Colchester, UK; 7 The BioRobotics Institute, Scuola Superiore Sant’Anna, Pisa, Italy

**Keywords:** Neurology, Electromyography - EMG, Biomedical engineering

## Abstract

Hand movement is controlled by a large number of muscles acting on multiple joints in the hand and forearm. In a forearm amputee the control of a hand prosthesis is traditionally depending on electromyography from the remaining forearm muscles. Technical improvements have made it possible to safely and routinely implant electrodes inside the muscles and record high-quality signals from individual muscles. In this study, we present a database of intramuscular EMG signals recorded with fine-wire electrodes alongside recordings of hand forces in an isometric setup and with the addition of spike-sorted metadata. Six forearm muscles were recorded from twelve able-bodied subjects and nine forearm muscles from two subjects. The fully automated recording protocol, based on command cues, comprised a variety of hand movements, including some requiring slowly increasing/decreasing force. The recorded data can be used to develop and test algorithms for control of a prosthetic hand. Assessment of the signals was done in both quantitative and qualitative manners.

## Abstract (summary)

**Table:** 

Measurement(s)	muscle electrophysiology trait • muscle contraction
Technology Type(s)	micro electrode • strain gauges
Factor Type(s)	age • muscle
Sample Characteristic - Organism	Homo sapiens

Machine-accessible metadata file describing the reported data: 10.6084/m9.figshare.11310011

## Background & Summary

Normal control of hand muscles is usually executed with little mental effort through the large number of control and feedback signals that bind together the central nervous system and the muscles^1,2^. Muscles controlling hand are located in the proximal parts of the hand and they have long, sometimes multiple tendons crossing several joints and sometimes acting on multiple fingers. Thus, control of hand movements requires a complex pattern of neural activation and inhibition of several muscles. The complexity of this system become obvious following amputation of a hand and forearm where central command and the proximal nerve signal remains operational but without end-effectors to complete the desired action.

Powered hand prosthetic devices offer the possibility to produce grasps upon user’s intention which is commonly detected by means of surface electromyography (EMG) resulting from the remaining muscles that are still interfaced with motoneurons^3^. This principle is employed by many state-of-the-art EMG-controlled prosthetic hands^4–6^, but when compared with the dexterity of normal hand, prosthetic devices are often restricted to producing only basic functions, such as closing/opening^7^. This limitation arises from the inability of the control algorithms to decode elaborate human intentions through EMG recorded on the skin surface. Although various computational methods have been suggested^8–13^, there is still no accurate and robust method for translating surface EMG into proficient proportional multi-degree-of-freedom hand control. This is mainly due to physical limitations of the acquired signal which contains superimposed activity of all the underlying muscle units. Because the electrical signals propagate through layers of biological tissue, the superficial muscles have a proportionally larger contribution to the EMG signal than the deeper ones^14–16^ (i.e. supinator). Additionally, when recorded on the skin surface, the EMG signal is prone to large distortions due to relative movement between the electrodes and the muscles as the arm moves.

One technique to obtain more informative EMG signals is to place electrodes close to, or even inside, the targeted muscles in order to selectively record activation patterns of multiple muscles independently. In this way, it is possible to derive relatively simple but intuitive methods that could link the muscle activity to the movement of the artificial hand. With technological advances^17–20^, surgical implantations of electrodes are becoming a reality for widespread use, thus enabling development of novel control strategies based on intramuscular EMG (iEMG)^21,22^. Having in mind that the main requirement of such control algorithm is high reliability related to interpreting user intent regardless of circumstances, it is clear that an algorithm should be thoroughly tested on various iEMG signals recorded on subjects during execution of common hand movements. The systematic way of designing appropriate multi-degree hand prosthesis controller usually relies on a relatively large collection of input signals obtained in a protocol similar to the intended use scenario. Therefore, a database of iEMG signals would be instrumental for any future development of control algorithms for hand prosthesis and for cross evaluation of different classification approaches. Unfortunately, compared to surface EMG, collecting iEMG requires a significantly more elaborate procedure which includes medical specialists and strictly controlled conditions. Thus, there are only a few previous studies that have focused on obtaining iEMG for the purpose of controlling a hand prosthesis^21,23–26^.

Here we present a database of iEMG signals recorded during the execution of basic hand movements and some of the most common hand gestures. The rationale behind this protocol is to enable prediction of a single degree of freedom (such as flexion/extension of individual fingers) but also to test the algorithm on synergistic hand gestures. The signals were recorded using fine-wire intramuscular electrodes positioned by a clinical neurophysiologist. The protocol included visual and audio cues presented to a subject in order to perform specific motor tasks. Simultaneously with the recording of iEMG, forces elicited by the fingers and wrist were recorded using a custom-made isometric force measurement device. The fact that multi-joint forces were accurately and synchronously recorded with iEMG is the major highlight of this database. Fourteen able-bodied subjects participated in the study divided into two protocols; the first focused on the muscles available within a short residual forearm; the second focused on fingers and thumb muscles. The database comprises 16 self-containing files that include iEMG signals, force signals and protocol descriptors.

## Methods

### Subjects

Fourteen male able-bodied volunteers aged between 25 and 57 years (mean 39 years) participated in the study. All subjects were right-handed and neurologically intact. The recordings were divided into two protocols and two subjects were included in both protocols, thus the final database contains sixteen files, eight for each protocol. All the subjects signed the informed consent, and the study was approved by the Regional Ethical Review Board in Lund, Sweden (Dnr 2017-297).

### Intramuscular EMG recordings

The intramuscular EMG signals were recorded using the Quattrocento (OT Bioelettronica, Torino, Italia) biomedical amplifier system. The system comprises a 400 channel amplifier, digitalization unit and preamplifiers with 5x gain for interfacing electrodes with the amplifier. All iEMG signals were amplified 150 times and sampled with 16-bit amplitude resolution at 10240 Hz. A hardware high-pass filter at 10 Hz and a low-pass filter at 4400 Hz were used during recordings. The intramuscular electrodes used in this study were paired fine-wire electrodes from Chalgren, Gilroy, USA. Each wire is 200 mm long and 0.051 mm in diameter, made of stainless steel with nylon insulation for improved visibility. Each pair of wires ends with a 2 mm bare wire (the insulation is offset to avoid short circuits). The positioning of the fine-wire electrodes was performed by a MD specialist in clinical neurophysiology using the guidelines from *Anatomical guide for the electromyographer: the limbs and trunk*^27^. The localization of the inserted wires was done by checking the movement of the guiding cannula while performing designated hand manoeuvre specified for the targeted muscle. The second check of the signal quality was done with the cannula still inserted by displaying iEMG signal in real-time while performing the hand manoeuvre. The final check of the signal was after the removal of cannula. Upon validation that the fine-wires were inside the targeted muscle, the wire leads and the preamplifier were taped to the lower arm to ensure low noise measurement. In addition, to verify the correct position of the electrodes a MD specialist in musculoskeletal radiology performed an ultrasound examination in two subjects (subjects 13 and 14), one per each recording protocol. The aim of ultrasound examination was to confirm that the needle tip was located within the targeted muscle after which the needle was retracted. The DICOM and JPEG images of the needle positions within the targeted muscles are provided with the rest of the recorded data. It should be noted that the ultrasound machine used for this study (EPIQ 7, Phillips, The Netherlands, with linear transducer L18-5 working on 9 MHz) was not able to detect the fine-wire ends due to their small size, so the position identification was done using the needle tip.

In this study we defined two measurement protocols, each targeting specific muscles which correspond to two different levels of forearm amputation, thus mimicking potential user scenarios of myoelectric prosthesis control. The first protocol, called short residual limb (SRL) protocol targeted the following muscles: flexor carpi radialis (FCR) – responsible for wrist flexion, extensor carpi radialis longus (ECR) – responsible for wrist extension, pronator teres (PT) – responsible for forearm pronation, flexor digitorum profundus (FDP) – responsible for flexion of fingers D2-D5, extensor digitorum communis (EDC) – responsible for extension of fingers D2-D5, and abductor pollicis longus (APL) – responsible for thumb abduction. The second protocol, called long residual limb (LRL) targeted the following muscles: flexor digitorum profundus (FDP), extensor digitorum communis (EDC), abductor pollicis longus (APL), flexor pollicis longus (FPL) – responsible for thumb flexion, extensor pollicis longus (EPL) – responsible for thumb extension, and extensor indicis proprius (EIP) – responsible for index finger (D2) extension. The splitting into two protocols, each focusing on one of the scenarios (SRL or LRL) encountered in amputees also reduced the chance of failed measurements due to, for example a subject experiencing vasovagal syncope (fainting) or a high level of pain or discomfort. The twelve subjects had six pairs of fine-wires inserted regardless of the protocol, while two subjects were recorded with nine electrodes that were later also divided into two subsets (SRL and LRL).

### Isometric force recording

Synchronously with the iEMG signal recording, hand forces were assessed using a custom-made force measurement device. The main reason for using the force/torque measurement device was to gather data of individual joint torques during the execution of predefined movements. The rationale for choosing an isometric setup was to simulate muscle behaviour in a forearm amputee and to limit muscle displacement that could impact mechanical stability of the inserted fine-wire electrodes. The device was designed in order to constrain the joints in the wrist and hand in a neutral posture approximately in the middle of the range of motion of the individual joints. In addition, this biomechanically favourable position enabled the generation of relatively large forces for both flexion and extension of individual joints. During the measurement, the device was firmly placed on the table with rubber patches preventing the device from slipping or accidental lifting. The subject’s forearm was supported by an adjustable bracket, and also, the subject was instructed to remain firmly seated throughout the measurement session to prevent compensatory movements. The force sensors’ outputs were conditioned to an output of 0–5 V, with the voltage proportional to the exerted force in the range ± 100 N. The sensors were factory calibrated by the manufacturer in order to have less than 1% full scale error. The linearity error was also validated after integrating force gauge sensors into the device. This was done to determine influence of the semi-rigid aluminium frame on the measured force. The resulting transfer function between readings of the reference force gauge and the sensor of the instrumented platform was highly linear throughout the operating range (R2 = 0.9999, RMSE = 0.010 42 V (0.41 N)). The analog signals from the force sensors were digitalized using NI-USB 6218 (National Instruments, Austin, Texas, USA) A/D with 16-bit amplitude resolution and sampling frequency of 200 Hz. A custom-made LabView (National Instruments, Austin, Texas, USA) program recorded force signals in a _.tdms file. At the onset of recording, the NI-USB 6218 card generated pulses that were routed to Quattrocento auxiliary input in order to synchronize the force and EMG data. The synchronization pulses had 5 V amplitude, 200 ms width and 0.5 Hz frequency and were also recorded with the NI card analog input to ensure synchronization in the case of any kind of delay related to the output of the NI device.

The detailed description of the measurement device and its error validation can be found in our previous publication^28^.

### Recording protocol

**Fig. 1:**
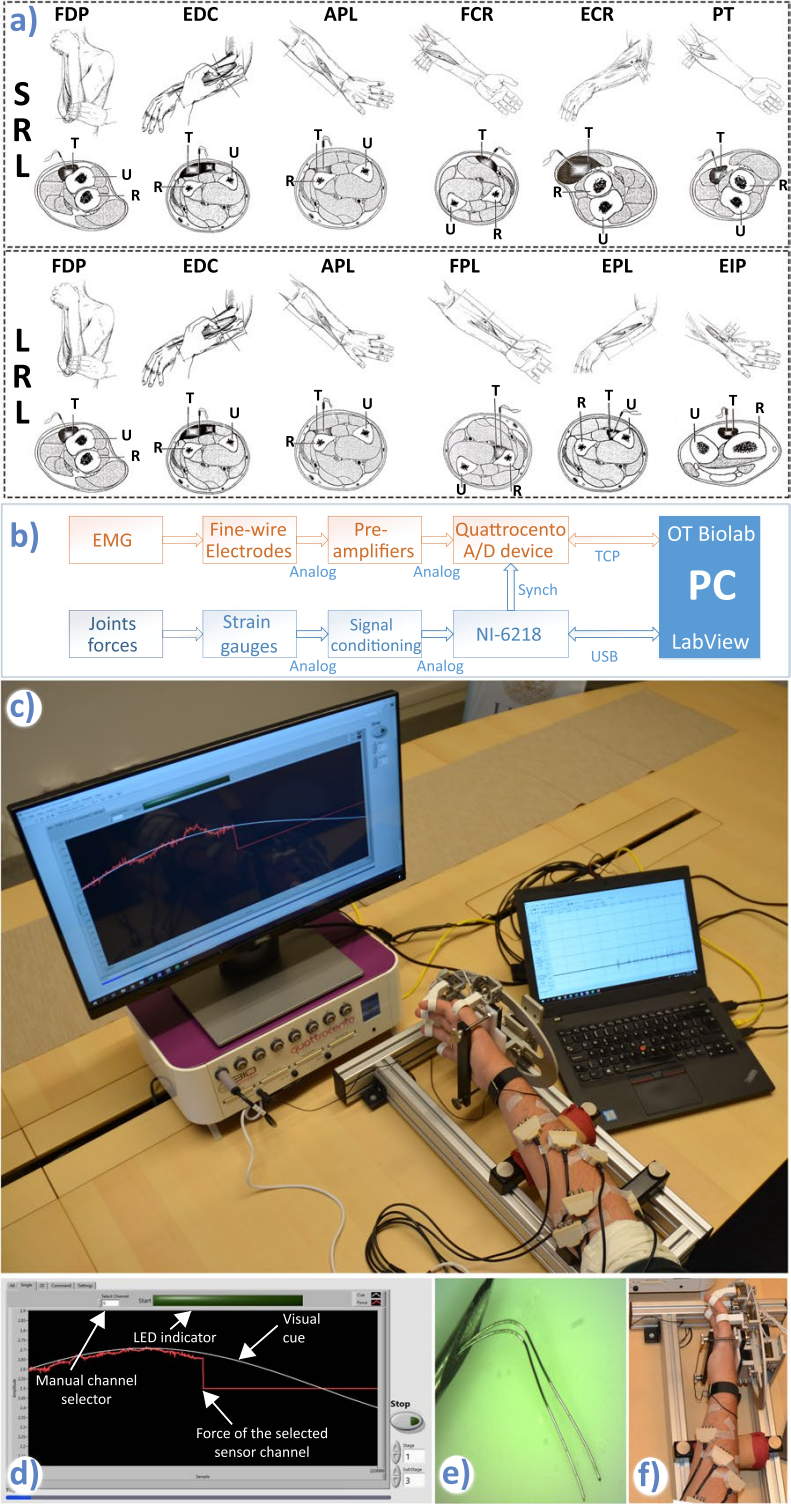
Experimental setup. (**a**) Targeted muscles for the short residual limb (SRL) and long residual limb (LRL) protocols. Upper rows of both protocols show manoeuvres preceding needle insertion and the insertion points. Bottom rows show locations of targeted muscles (T), ulna (U) and radius (R) in the forearm cross sections at the needles insertion points. Modified from Perotto *et al*.^27^. Courtesy of Charles C Thomas Publisher, Ltd., Springfield, Illinois. (**b**) Signal measurement chain showing two separate systems for acquisition of EMG and joint forces. The hardware synchronization was implemented as a digital line between the NI card and the Quattrocento device. (**c**) Measurement setup: Force/torque measurement apparatus, preamplifiers, visual cue and measurement flow control, Quattrocento amplifier and OT BioLab software for real-time monitoring of the iEMG signals. (**d**) Screenshot of the LabVIEW GUI during the sine-tracking task. (**e**) Fine-wire electrode tips. (**f**) Position of the hand within the force measurement device.

As stated before, two measurement protocols were designed for this study, with the only difference being the selection of targeted muscles. Thus, besides the recorded muscles, the protocol was the same in both cases. At the beginning of the recording session the subject was informed about all the aspects including possible risks after which he signed the informed consent. In the next stage, the isometric force measurement device was configured to fit the subject’s hand. This was followed by a familiarization period (lasting approximately 30 minutes) during which the subject got accustomed to the required hand movements, software and cues. Upon completing the familiarization period, the neurophysiologist placed the electrodes. If a subject experienced high level of pain or nausea the measurement was paused and the continuation was evaluated by the neurophysiologist. Two of the volunteers fainted during the electrode insertion procedure and did not participate in the recordings. Additionally, one subject experienced excessive pain from the electrode positioned in pronator teres muscle which had to be removed. Nevertheless, in this case, the recording was performed with the other five electrodes. Although occasionally experiencing pain or nausea, other subjects had all the electrodes in designated muscles. After securing all wires and preamplifiers with the adhesion tape, the subject was instructed to place his hand in the force measurement device and sit comfortably (see Fig. 1).

The whole measurement protocol was controlled and guided automatically (without the need of a manual protocol control or an instructor) by the custom-made software developed in LabVIEW. Before each task, a short written description was presented on the screen, followed by the onset indicator comprising a large green light indicator (visual cue) and a beeping sound (auditory cue). This “go cue” was only relevant for maximal voluntary contraction (MVC) and grasping stages of the measurement as the rest of the protocol stages were focusing on sine tracking. During the measurement, selected forces were presented to the subjects, e.g. while performing index finger movement, only the index finger force was shown. During any movement, a manual override of a presented cue was possible upon subject’s request, e.g. decreasing/increasing visual cue force. As the whole process was automatic, codes for individual stages were generated and recorded alongside the force signals. The automated protocol executed experimental substages in the same order as presented in Table 1. During the measurements, a clinical neurophysiologist was observing the iEMG signals in real-time.

**Table 1:** The list of different movements and their codes in the database files. The similar table can be found within each database entry (cell “.Movements”). In this table x could be 0 or 1, where 1 denotes duration1.1 of the movement cue and 0 rest period within the protocol substage.

Movement		Sub stage	Code
1. Single degree of freedom (DoF) Tasks	Index finger: flexion-extension	MVC Flexion	1.1x
		MVC Extension	1.2x
		Track sine cue	1.3
	Middle finger: flexion-extension	MVC Flexion	2.1x
		MVC Extension	2.2x
		Track sine cue	2.3
	Ring finger: flexion-extension	MVC Flexion	3.1x
		MVC Extension	3.2x
		Track sine cue	3.3
	Little finger: flexion-extension	MVC Flexion	4.1x
		MVC Extension	4.2x
		Track sine cue	4.3
	Thumb: flexion-extension	MVC Flexion	5.1x
		MVC Extension	5.2x
		Track sine cue	5.3
	Thumb: adduction-abduction	MVC Adduction	6.1x
		MVC Abduction	6.2x
		Track sine cue	6.3
	Wrist: flexion-extension	MVC Flexion	7.1x
		MVC Extension	7.2x
		Track sine cue	7.3
	Wrist: supination-pronation	MVC Supination	8.1x
		MVC Pronation	8.2x
		Track sine cue	8.3
2. Single joint-multiple DoFs tasks (joysticks)	Thumb: Joystick flexion-extension-adduction-abduction	Track circular cue	9.1
	Wrist: Joystick flexion-extension-supination-pronation	Track circular cue	10.1
3. Double DoF task (Trumpet test)	“Touch index finger with the thumb”	Track sine cue	11.1
	“Touch middle finger with the thumb”	Track sine cue	12.1
	“Touch ring finger with the thumb”	Track sine cue	13.1
	“Touch little finger with the thumb”	Track sine cue	14.1
4. Synergistic movements	All fingers flexion (without thumb)	4 repetitions	15.1x
	All fingers extension	4 repetitions	16.1x
	Palmar grasp	4 repetitions	17.1x
	Pronation followed by Palmar grasp	4 repetitions	18.1x
	Pointing: index-extend, digits 3–5 flex	4 repetitions	19.1x
	3-digit pinch	4 repetitions	20.1x
	3-digit pinch with pronation	4 repetitions	21.1x
	Key grasp followed with pronation	4 repetitions	22.1x

The system setup for the basic movements’ protocol (Single DoF Tasks) included a MVC of individual joints (codes x.1 and x.2 for x in [1,8]) and a torque tracking task (code x.3 for x in [1,8]). For the MVC task, the subjects were instructed to perform one strong contraction using only the specified joints. The onset and duration (5 s) were guided by the onscreen indicator and a beeping sound. These periods are labelled in the recorded files with increments of 0.01 on the base movement codes. For example, code 3.11 denotes actual MVC contraction cue of the ring finger while 3.10 is rest/preparation period within the ring finger MVC sub stage. The MVC test was performed to assess maximal joint force/torque (used in cue test as the reference) and to record muscle contraction with the maximal number of active fibres. In the tracking tasks, the subject was asked to produce force/torque that matched the cue presented on the screen. Sinusoidal waveforms were provided as visual cues to estimate gradual force increase. The rationale behind the sinusoidal tracking task was to provide iEMG data and force data for proportional control of a hand prosthesis. The repetition frequency was set to 0.1 Hz to enable a gradual and controllable force increase. In addition to the basic movements (individual fingers and wrist flexion-extension, abduction-adduction and pronation-supination) a set of single joint 2DoF (code x.1 for x in [9,10]), multi-joint 2DoF (code x.1 for x in [11,14]) and synergistic (code x.1 for x in [15,22]) hand and wrist movements were included in the protocol. Both single and multi-joint 2DoF movements were based on trajectory tracking cues. In the case of single joint movements, the subject was tracking a 2D trajectory (circle) on the screen, where the circle radius was calculated as the lesser of two values from the corresponding single DoF sub stages. For example, for thumb joystick sub stage (9.1) the circle radius was calculated using the smaller of the two amplitudes used for sub stages 5.3 and 6.3. The synergistic movements were defined as 4 repetitions of movements with self-chosen force and timings governed by both a visual and an audio cue. The subjects were instructed to execute the synergistic multi-joint movements synchronously (all at once) except for the two movements that included the word “followed” in the movement description (see movements 18 and 22 in Table 1). In these two cases, the subjects were instructed to first complete the first movement (i.e. for stage 18: pronation) and then start the second movement (i.e. for stage 18: palmar grasp). To make it clear, we included this explanation within the text. The labeling of synergistic movement cues was done similarly to the labeling of the movement cue implemented for single DoF MVC sub stages. For example, 3-digit pinch movement cue was labeled with 0.01 increment on the base movement code resulting in 20.11, while the rest between repetitions was labeled with 20.10. The chosen movements were foreseen as the most common gestures and grasps that a user will frequently perform with a hand prosthesis^29–31^. The recording lasted for 30 minutes. A mock-up video (iEMG_movements.mp4) demonstrating the hand gestures in free-movement conditions is provided within the same repository as the recorded data.

### Data pre-processing

The iEMG data recorded with OT BioLab was imported to Matlab (MathWorks, Natick, Massachusetts, USA). The second data file (_.tdms) containing force measurement, synchronization signals, tracking cue, the stage label, channel labels and time stamps recorded with the NI-6218 and LabVIEW software was also imported in Matlab. The first step of the data processing was to remove 50 Hz noise and its harmonics from the iEMG data. Although iEMG was recorded with an amplifier with 50 Hz suppression circuitry, the recorded data still contained some pronounced spectral components at 50 Hz and some of the harmonics. To reduce the noise, a filter bank comprising narrow notch filters centred at 50 Hz and harmonic values up to 5 kHz with 2 Hz width was constructed. The applied filter was customized for each recording to minimize spectral distortion. To conserve synchronization between channels, zero phase filters (Matlab command: filtfilt) were used. The next step in the data processing was to detect synchronization pulses in both files. This was done by numerically differentiating the 1D synchronization signal (Matlab command: diff) to emphasize pulse edges. Using the differentiated signal, the detection of the first pulse leading edge and the last pulse trailing edge in both files was performed. As a check-up, the count of all synchronization pulses was compared to verify that both files contain the same data. To prepare the data for the joint database, an interpolation of the force data gathered using LabVIEW was done to accommodate the Quattrocento iEMG data sampling rate (Matlab command: interp1).

Besides raw data, motor unit action potentials (MUAPs) detected and sorted from the recorded iEMG are provided as metadata. MUAP sorting was performed using an algorithm based on prior work by the authors^32^, which assigns MUAPs to the most similar waveform template in a set. To assess similarity between the MUAPs and each waveform template, the MUAPs were first aligned to the template using the lag that maximizes the cross-correlation between them, and then the following two criteria were evaluated: a) correlation coefficient between the MUAP and waveform template greater than 0.9; b) mean square difference between the MUAP and the waveform template smaller than half of the power of the waveform template. The spike sorting algorithm was executed three times, in which all motor unit action potentials (initially detected via a threshold) were processed in chronological order. In the first iteration, if the current MUAP was similar to any template already in the set, that template was updated to account for the new MUAP; otherwise, a new template was created. In the second iteration, all detected MUAP were re-processed, and existing templates were updated, but no new templates were created. In the last iteration, MUAPs were compared with each template and labelled as belonging to their best match, provided the above criteria were met, otherwise the MUAP was labelled as unknown. At the end of the first two iterations, the templates that had less than 5% of MUAPs with respect to the total number of MUAPs were discarded.

As the database is primarily recoded for the purpose of deriving and testing of different control algorithms for prosthetic hands, an iEMG signal feature was also calculated using raw signals. To provide signals for the benchmarking of novel algorithms, envelopes of the recorded iEMG signals were extracted and provided as metadata. The Root Mean Square (RMS), as one of the most common EMG signal features, was selected to represent the signal envelope. In the metadata associated with the main iEMG database, the RMS was calculated using 250 ms wide window which was shifted in steps of one sample. The sliding window was centred over the current sample, resulting in zero phase shift between the raw signal and its envelope. It should be noted that this processing does not satisfy the condition of causality that is present in real-time applications where the value of the current signal feature could be calculated using only previous samples. Nevertheless, the zero-phase shifted envelope is provided with the data as a measure of an “ideal” signal feature, and it could be transposed into a realistic envelope by shifting the whole signal by half of the window size (125 ms which equals 1280 samples). The envelope signals were not normalized.

## Data Records

The database associated with this paper can be found at figshare^33^.

The data associated with each recording was stored in two files: one containing all the recorded signals and the experimental setup information (channels labels, movement codes and sampling rate), and another containing the spike-sorted metadata.

The pre-processed signals were bundled together and stored as Matlab structure files. The filename of each Matlab file consist of the following string: “FW” indicating the fine-wire recording, “SRL” or “LRL” depicting protocol and “Sxx” indicating subject coded name. The Matlab structure of each file contains the following elements:

- .Data
- .Channels
- .Movements
- .fs

The *Data* element holds all iEMG channels, all force channels, movement code and actual cue level presented to the subject (for sine tracking task). The iEMG signals are given in mV. The forces are presented in their unprocessed form; they are given in V while the transfer function between sensor analog output and the force is following:

$${\rm{force}}={\rm{analog}}\text{\_}{\rm{voltage}}\ast 40-100\left[{\rm{N}}\right]$$

The *Channels* element contains channel labels that correspond to columns of the Data element. The *Movements* element of the file structure contains a list of hand movements/gestures alongside their codes that are within *Data* element (similar to Table 1). Finally, the *fs* element depicts the sampling frequency of the data. The list of files within the database is given in Table 2.

**Table 2:** Data files and age of the subjects.

Subject	Protocol	File name	Age
1	SRL	FW_ SRL _S1.mat	33
2	SRL	FW_ SRL _S2.mat	47
3	SRL	FW_ SRL _S3.mat	34
4	LRL	FW_ LRL _S4.mat	42
5	SRL	FW_ SRL _S5.mat	42
5	LRL	FW_ LRL _S6.mat	42
6	SRL	FW_ SRL _S7.mat	35
6	LRL	FW_ LRL _S8.mat	35
7	LRL	FW_ LRL _S9.mat	57
8	SRL	FW_ SRL _S10.mat	41
9	LRL	FW_ LRL _S11.mat	40
10	SRL	FW_ SRL _S12.mat	33
11	LRL	FW_ LRL _S13.mat	42
12	LRL	FW_ LRL _S14.mat	35
13	SRL	FW_ SRL _S15.mat	25
14	LRL	FW_ SRL _S16.mat	43

**Fig. 2:**
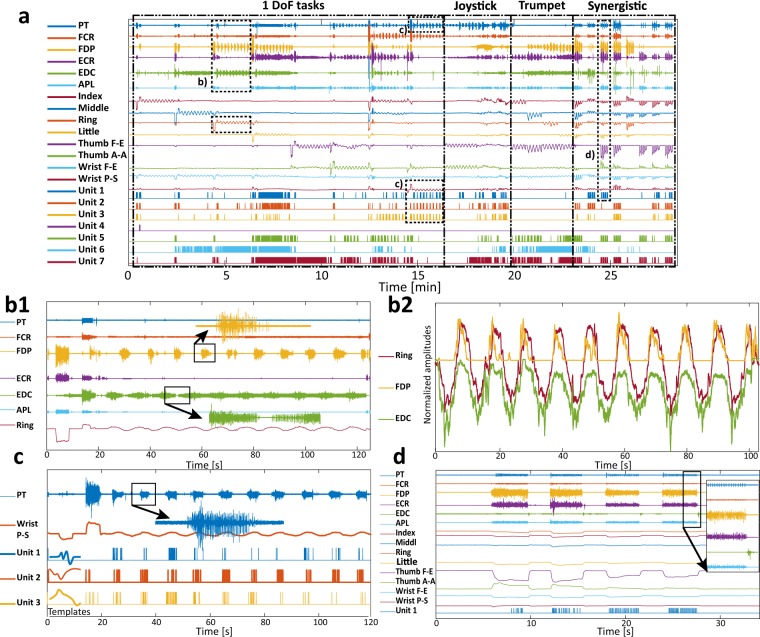
Sample of recorded data, subject 7. (**a**) Recorded signals in one full session: 1–6. iEMG channels (pronator teres – PT, flexor carpi radialis – FCR, flexor digitorum profundus – FDP, extensor carpi radialis – ECR, extensor digitorum communis – EDC, abductor pollicis longus – APL), 7–14 Strain gauges’ outputs. Additionally, activity of 7 muscle units (Unit 1–7) were joined with the recorded signals. The movement groups are separated with dashed lines. To provide more information regarding the recorded signals 4 cases are extracted from this recording and shown in the smaller time window. (**b1**) This subfigure highlights a single DoF movement. The ring finger force is notably correlated with the FDP and the EDC activities. It could be also noted that during force modulation both firing rate and recruitment of these muscles are changing. (**b2**) This subfigure illustrates estimation of ring finger force using RMS features of FDP and EDC channels. From the shapes of the time domain signals it could be noted that activity of two antagonistic muscles is reflected in finger force. This correlation is prominent in global trends (increasing/decreasing) of the finger force (increase/decrease), but also in small disturbances of the finger force that are present in iEMG RMS features (**c**) This subfigure focuses also on a single DoF movement but, besides raw muscle activity the figure also shows extracted spike trains. In the case of activity in the PT, it is interesting that for small forces only single muscle unit was picked-up by the fine-wires so the force modulation is reflected in the firing rate of that MU. The lower part of the subfigure shows three spike templates. (**d**) This subfigure depicts a synergistic movement that comprises the strong contraction of the majority of the targeted muscles. The aim of this subfigure is to show differences between channels in terms of iEMG content. While in some channels there is only one (or several) MU active, other channels are recording an abundance of MU-s resulting in a signal shape that resembles superficial EMG.

One should note that subjects 5 and 6 underwent both protocols at the same time, effectively having nine pairs of fine-wires positioned in all targeted muscles involved in both protocols while executing movements only once. This gives opportunity to researchers to merge these recordings in order to evaluate synergies and activation patterns of all nine muscles. A sample recording of iEMG and force channels from the file FW_SRL_S7.mat is shown in Fig. 2.

In addition to recorded signals, a supplementary set of 16 Matlab files containing spike sorted data (one per participant, named spikesort_sXX.mat, where XX is the subject identifier) is available at the same repository. These files contain four elements:

- .samples: contains as many cells as spike trains that were obtained from that participant. Each of the cells represents a spike train. The numbers are the samples at which the MUAPs occur.
- .times is a different way of observing the ‘samples’ data. In this instance, the numbers in each cell are the time stamps of the MUAPs, instead of the samples. That is, each number is the corresponding value from .samples divided by the sampling frequency.
- .template: contains action MUAP waveform templates.
- .fs is the sampling frequency.

Another set of 16 Matlab files containing iEMG signal envelopes is also provided in the same repository. These files contain six envelope signals each in the same order as in raw iEMG signal files. The lengths of envelopes are same as the raw iEMG signals that were used to extract these signal features.

Finally, to facilitate simpler reuse of the iEMG signals database, a table comprising pairs of iEMG channels and force channels was derived and also provided as metadata. The table was constructed based on qualitative evaluation of signals quality done by the neurophysiologist during which iEMG signal properties (onset and modulation) were compared with the forces exerted by the hand joints. The table contains:

- List and order of iEMG channels for each subject.
- Pairs of iEMG channels and force channels. This sub-table also comprises code of the movement during which maximal correlation between these two channels was observed. In example, for subject 7, FDP channel (column 3 in the file container) pairs with little finger force channel (column 12 in the file container) during little finger movement (movement code 4). In addition, to enable direct comparison between iEMG features and forces, this sub-table contains the *sign* that indicates the force direction that corresponds to the iEMG channel activity. In example, for subject 7, the little finger force gets negative (flexion) when FDP channel has iEMG activity, thus the *sign* is −1. The *sign* parameter is the consequence of the bidirectional force transducers that were measuring both flexions and extensions (adductions/abductions and pronations/supinations) of individual joint.
- Quality of iEMG signals. This sub-table is also shown in Table 3. It should be noted that the pairing was done regardless of the signal quality.

**Table 3:** Cross-correlation of different iEMG channels. The values shown in the table are median among all subjects with 5^th^ and 95^th^ quartile in brackets.

1	**1** (1; 1)					
2	**0**.**007** (0.005; 0.051)	**1** (1; 1)				
3	**0**.**007** (0.004; 0.011)	**0**.**006** (0.005; 0.022)	**1** (1; 1)			
4	**0**.**006** (0.004; 0.023)	**0**.**006** (0.004; 0.064)	**0**.**006** (0.004; 0.034)	**1** (1; 1)		
5	**0**.**006** (0.005; 0.044)	**0**.**006** (0.004; 0.027)	**0**.**007** (0.004; 0.030)	**0**.**006** (0.004; 0.033)	**1** (1; 1)	
6	**0**.**008** (0.005; 0.073)	**0**.**006** (0.004; 0.041)	**0**.**007** (0.005; 0.027)	**0**.**013** (0.005; 0.106)	**0**.**009** (0.004; 0.066)	**1** (1; 1)
	1	2	3	4	5	6

## Technical Validation

The technical validation of the recorded data was performed in two ways: qualitative and quantitative. In the qualitative analysis, the neurophysiologist reviewed the individual iEMG signals. This evaluation process comprised the following examinations:

- iEMG in time domain. This part of the evaluation focused on muscle unit action potentials (MUAP) shape and frequency. The iEMG was marked as proper if the signal contained clearly differentiated inactive and active segments (with and without muscle activity), and active segments with MUAPs of the physiological amplitude, duration and frequency.
- iEMG with respect to the performed movements and exerted forces. This evaluation aimed at comparing activity of the recorded muscle and the forces associated with that specific muscle. The iEMG signal was marked as proper if the activation of the muscle corresponded to the recorded force measured on the hand joint actuated by the examined muscle.
- iEMG channel with respect to other iEMG channels. This evaluation checked if there was significant overlap of activation between two or more recording sites, indicating that one or both fine-wires of the channel had migrated to a neighbouring muscle. The iEMG signal was marked as proper if the activations were not synchronous over the whole recording.

**Fig. 3:**
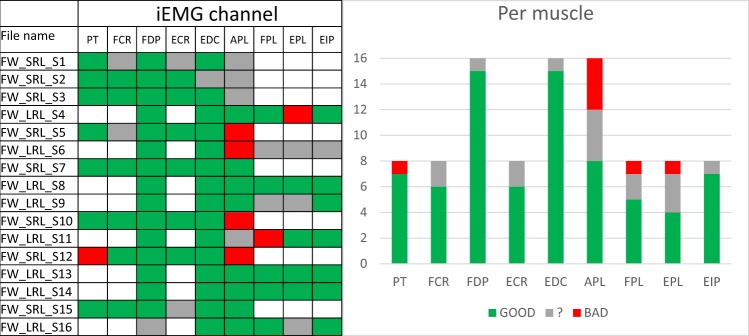
Qualitative assessment of the database signals. For each database file all signals were analyzed individually. iEMG signals/channels considered “good” are represented with green, “questionable” with grey and “bad” with red color (left). Qualitative analysis of recorded iEMG channels presented in absolute numbers (right). iEMG signals/channels considered “good” are represented with green, questionable (“?”) with grey and “bad” with red colour.

Only if the neurophysiologist marked all three indicators as proper the signal was considered as “good”. If there was no clear decision in any of the examinations the signal was considered as “questionable”, finally, if there was clear negative decision in any of the examinations the signal was considered as “bad”. The results from the qualitative assessment of the database signals are shown in Fig. 3.

When added together, the qualitative evaluation of the recorded iEMG signals show that 76% of channels were “good”, 17% “questionable” and 7% “bad”. To further identify errors during the experiment protocol, the qualitative evaluation was done also on individual channel/muscle basis. This analysis revealed that most of the “bad” channels were associated with thumb muscles (primarily APL), as shown in Fig. 3. It is important to note that for the two subjects (labeled as FW_SRL_S15 and FW_LRL_S16) who had their electrode positions verified using ultrasound, the number of questionable channels had the same ratio as for other recordings that were done without the ultrasound guidance.

The fact that measurement of iEMG on some channels/muscles resulted in several unsuccessful recordings could be explained by different reasons such as:

- Relatively small muscles. The thumb muscles are quite small in size making it difficult for the wires to remain anchored in the muscle. They are also positioned in vicinity of some larger forearm muscles, so any, even small, displacement of wire ends after the initial insertion could lead to significant crosstalk between muscles.
- Electrode placement protocol. The initial protocol defined the sequence of electrode insertions to start with the most distal and end with the most proximal muscle sites. This practically meant that the thumb muscles were targeted first. Consequently, as the electrode placements for the proximal muscles required maximal pronation/supination manoeuvres, as described in the “Anatomical guide for the electromyographer”^27^, there was a risk that some of these movements could displace a fine-wire from the small and deep muscle.

The one instance of PT muscle recording being “bad” was due to excessive pain experienced by the volunteer during the recording, which led to removal of that electrode.

In the quantitative analysis we checked two parameters of the recorded iEMG signals: cross-correlation between channels and the signal spectrum. The calculation of cross-correlation coefficients was done after offline notch filtering (Matlab function: *xcorr()*). The results of the cross-correlation calculation are shown in Table 3. The aim of Table 3 is to demonstrate the extremely high selectivity that fine-wire electrodes enable. Additionally, a low cross-correlation between channels shows that the common noise was successfully removed through amplifier design and offline filtering.

It should be noted that the cross-correlation coefficients between channels are quite low, even though qualitative evaluation done by the neurophysiologist concluded that some of the recorded iEMG channels were not recording the targeted muscle. This low cross-correlation indicates that, despite some of the electrodes recorded from the same muscle, due to their small exposed surface, they captured activity from different muscle portions whose activities are not synchronous.

**Fig. 4:**
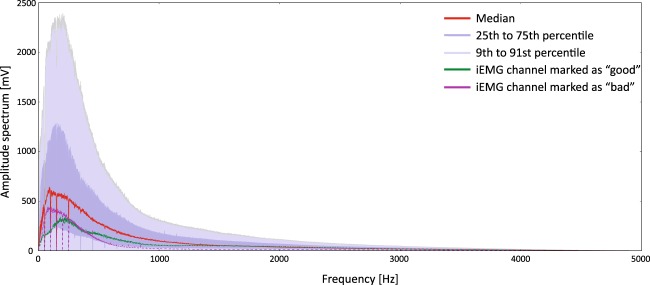
Cumulative representation of iEMG signals spectra. The red line represents median, dark blue represent area between 25^th^ and 75^th^ percentile and light blue represents are between 9^th^ and 91^st^ percentile of all the subjects, all channels and all movements. The figure also comprises samples of the amplitude spectrums from a “good” (green line) iEMG channel (subject 7, FCR muscle) and a “bad” (magenta line) channel (subject 11, FPL muscle).

The second quantitative test included evaluation of the iEMG signal spectrums. The cumulative plot of all iEMG signals is shown in Fig. 4.

It should be further noted that the median and quartiles of the signals spectrums closely match the shape reported in the literature^3^. The discontinuities within the spectrum are due to offline notch filters that removed power line interference. In addition, the dispersion of the signal spectrum amplitudes is relatively high which is a consequence of the fine-wire recording methodology. Specifically, there is no routine for precisely controlling free wire ends during or after inserting which results in wire ends having a variety of distances and orientations between them for different muscles and subjects. Consequently, depending on wire ends distances and orientations (along or across the muscle) there was a large variation between iEMG amplitudes on different channels.

In addition to the presented measures of the iEMG signal quality, we also utilized some of the well-known computational methods based on iEMG for the estimation of muscle force^21^. But, as the focus of this study was on the recording protocol and obtained signals, detailed information regarding the force estimation method and the results could be found in the previous publication from the same authors^28^.

## Data Availability

The signal recording was performed using two programs in parallel: OT BioLab version 2.0.6254 available at www.otbioelecttronica.com for recording iEMG and synchronization signals, and the custom recording software developed in LabVIEW 2016 for force signals recording, generating synchronization pulses, visualizing forces and generating commands and cues. Data post-processing was done in Matlab. All custom codes are available by contacting the corresponding author.
